# Supplementary figures and images for: Triglyceride-glucose index in the prediction of new-onset arthritis in the general population aged over 45: the first longitudinal evidence from CHARLS
Source: Lipids Health Dis. 2024 Mar 13;23:79. doi: 10.1186/s12944-024-02070-8 (PMC10936084; doi:10.1186/s12944-024-02070-8)

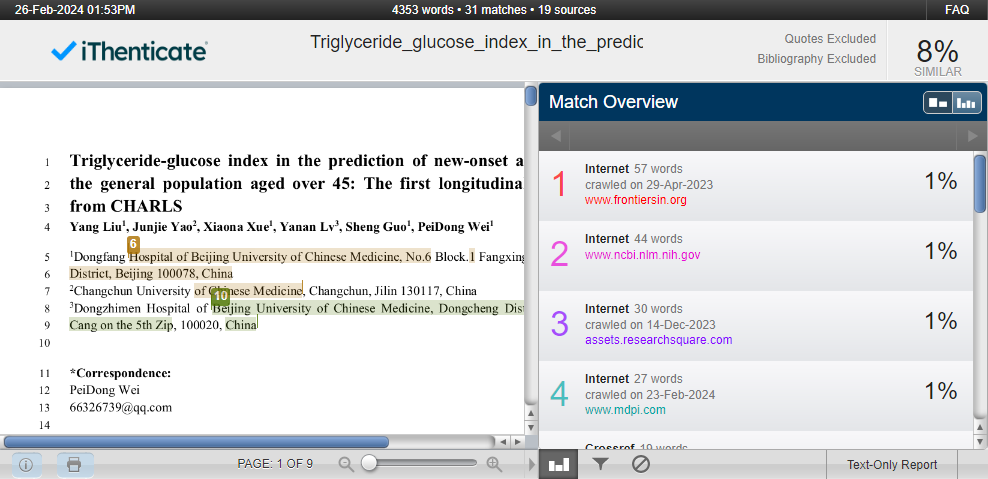

Supplement: Supplementary file 1 — Supplementary Material 1 [file 12944_2024_2070_MOESM1_ESM.png]
