# Supplementary material for: Triglyceride-glucose index in the prediction of new-onset arthritis in the general population aged over 45: the first longitudinal evidence from CHARLS
Source: Lipids Health Dis. 2024 Mar 13;23:79. doi: 10.1186/s12944-024-02070-8 (PMC10936084; doi:10.1186/s12944-024-02070-8)
Supplement: Supplementary file 2 — Supplementary Material 2 [file 12944_2024_2070_MOESM2_ESM.pdf]

Triglyceride_glucose_index_in_the_prediction_of_new_onset_arthritis_in_the_general_population_aged_over_45_The_first_longitudinal_evidence_from_CHARLS

WORD COUNT	4353	TIME SUBMITTED	26-FEB-2024 01:53PM
		PAPER ID	107142894

Triglyceride-glucose index in the prediction of new-onset arthritis in the general population aged over 45: The first longitudinal evidence from CHARLS

Yang Liu[1], Junjie Yao[2], Xiaona Xue[1], Yanan Lv[3], Sheng Guo[1], PeiDong Wei[1]

[1]Dongfang Hospital of Beijing University of Chinese Medicine, No.6 Block.1 Fangxingyuan, Fengtai District, Beijing 100078, China
[2]Changchun University of Chinese Medicine, Changchun, Jilin 130117, China
[3]Dongzhimen Hospital of Beijing University of Chinese Medicine, Dongcheng District, Hai Yun Cang on the 5th Zip, 100020, China

***Correspondence:**
PeiDong Wei
66326739@qq.com

Abstract

Objective

Insulin resistance (IR) imposes a significant burden on inflammatory diseases, and the triglyceride-glucose (TyG) index, which is an easily accessible indicator for detecting IR, holds great application potential in predicting the risk of arthritis. The aim of this study is to analyze the association between the TyG index and the risk of new-onset arthritis in the common population aged over 45 using a prospective cohort study design.

Method

This population-based cohort study involved 4418 participants from the China Health and Retirement Longitudinal Study (from Wave 1 to Wave 4). Multivariate logistic regression models were employed to investigate the association between the TyG index and new-onset arthritis, and RCS analyses were used to investigate potential non-linear relationships. Moreover, decision trees were utilized to identify high-risk populations for incident arthritis.

Result

Throughout a 7-year follow-up interval, it was found that 396 participants (8.96%) developed arthritis. The last TyG index quartile group (Q4) presented the highest risk of arthritis (OR, 1.39; 95% CI, 1.01, 1.91). No dose-response relationship between the TyG index and new-onset arthritis was identified ($P_{overall}$=0.068, $P_{non\text{-}linear}$=0.203). In the stratified analysis, we observed BMI ranging from 18.5 to 24 exhibited a heightened susceptibility to the adverse effects of the TyG index on the risk of developing arthritis (*P* for interaction =0.035).

Conclusion

The TyG index can be used as an independent risk indicator for predicting the start of new-onset arthritis within individuals aged 45 and above within the general population. Improving glucose and lipid metabolism, along with insulin resistance, may play a big part in improving the primary prevention of arthritis.

Keywords: Arthritis, Triglyceride glucose index, The China Health and Retirement Longitudinal Study (CHARLS), Insulin resistance, Cohort study.

1. Introduction

Arthritis, a degenerative inflammatory disease associated with ageing, is defined by two predominant subtypes: osteoarthritis (OA) and rheumatoid arthritis (RA). These conditions themselves are causing a burden on health due to demographic shifts towards an older age group [1]. Arthritis not only induces health consequences such as joint damage, pain, and impaired mobility but also exerts a burden on various physiological systems including the cardiovascular, renal, and nervous. Severe complications may even escalate mortality rates [2]. Nevertheless, despite the escalating medical expenses associated with arthritis treatment, there has been no substantial enhancement in the quality of life for patients who continue to endure pain, disability, and psychological distress [3, 4].

Insulin resistance (IR) has emerged as a significant risk factor for the onset of arthritis, representing a key component of metabolic syndrome that contributes to disease progression from multiple perspectives. Specifically, it elicits systemic inflammation and immune dysfunction by triggering the secretion of pro-inflammatory cytokines like tumour necrosis factor-alpha (TNFα) and interleukins (IL) [5, 6]. Therefore, regular assessment of IR may facilitate early detection of arthritis risk and control disease advancement. The gold standard for assessing insulin resistance (IR) is the widely acknowledged Hyperinsulinemic euglycemic clamp (HEC) technique. However, due to its invasive nature, high cost, and time-consuming process, HEC is difficult for subjects to accept, which makes it impractical for large-scale clinical research [7]. Homeostatic Model Assessment of Insulin Resistance (HOMA-IR) has gained widespread use in practice for its simplicity and non-invasive nature. However, its ability to quantify insulin resistance values becomes limited when the pancreatic beta-cell function is impaired or depleted, making it more suitable for research where IR is a secondary focus [8]. In 2008, the Triglyceride-Glucose (TyG) index was presented as a straightforward alternative indicator of IR. It utilizes fasting triglyceride (TG) and glucose quantitative values obtained from routine biochemical tests to provide a highly sensitive and specific measure of IR [9]. Moreover, the TyG index partially compensates for the limitations in assessing pancreatic β cell dysfunction that exists with HOMA-IR [10]. Further research has demonstrated that the TyG index exhibits superior performance compared to HOMA-IR in forecasting diseases highly associated with IR such as metabolic syndrome and diabetes [11, 12].

Recent cross-sectional studies have indicated an association between a higher TyG index and an elevated risk of arthritis [13]. However, the design limitations of cross-sectional studies restrict the ability to infer causality between the two factors. Therefore, this study aims to assess the causal relationship between the levels of the TyG index and the occurrence of arthritis, using longitudinal data from the China Health and Retirement Longitudinal Study (CHARLS).

2. Method

2.1 Study population

The CHARLS project's goal is to assemble a high-quality microdata set from a representative cohort aged 45 and above, to facilitate interdisciplinary research on population ageing in China. In 2011, a multi-stage probability-proportionality of size sampling method was used to conduct the CHARLS national baseline survey. The sample included more than 17,000 individuals residing in

approximately 10,000 households distributed across 150 counties within 28 provinces. The CHARLS survey is ongoing, with checkups every 2 to 3 years. Respondents were personally interviewed in their residences using computer-assisted face-to-face interviews. The survey asked about the respondent's and his or her household's basic demographics, transfers among household members, the respondent's health status, health care and insurance, employment, income, expenditures, and assets. Notably, in both 2011 and 2018, experts collected venous blood samples from individuals who had observed a fasting period of at least 12 hours without consuming any food or beverages. Adhering to rigorous standards, all biomedical procedures were executed by accredited professionals. These specimens, maintained at an optimal 4°C, were promptly dispatched to the Beijing Central Laboratory (You'anmen Clinical Laboratory Center, Capital Medical University) for advanced diagnostic assessments. Leveraging enzyme colorimetry, precise measurements were ascertained for glucose concentrations, TG, low-density lipoprotein cholesterol (LDL-C), and high-density lipoprotein cholesterol (HDL-C).

Figure 1 presents the flowchart detailing the participant selection process. Of the participants in the 2011 baseline survey, 17,707 individuals completed both the physical examinations and the questionnaire assessments. Among these participants, participants were subsequently excluded from the study according to the following specific standards: age below 45 years (423 individuals), history of lipid-lowering and glucose-lowering medication use (1416 individuals), incomplete TyG data (5530 individuals), missing arthritis information for both 2011 and 2018 (5250 individuals) and diagnosis of arthritis at baseline 670 individuals). The history of lipid-lowering and glucose-lowering medication use was obtained based on self-reporting by the participants. In the CHARLS questionnaire, the relevant question asked was, "Are you now taking any of the following treatments to treat [dyslipidemia or diabetes or hyperglycemia] or its complications (Check all that apply)?" Options included taking Western modern medicine, taking Chinese traditional medicine, or other treatments.

The Ethical Review Board of Peking University initially approved the CHARLS in 2008 (IRB0000105211,015). This study methodology adhered to all pertinent standards and recommendations set forth by CHARLS. Before participation, each volunteer provided informed consent by completing a consent form.

2.2 Assessment of the TyG index

The following formula was used to determine the TyG index: ln [triglyceride concentration (mg/dL) × fasting blood glucose concentration (mg/dL)/2].

2.3 Assessment of new-onset arthritis

The diagnosis of new-onset arthritis was based on self-reported data. When the interviewer asked, "Have you been diagnosed with arthritis by a doctor?" and the respondents answered "Yes," they were classified as arthritis patients. Subjects who had arthritis in 2011 were excluded, and if the patient was diagnosed with arthritis after that until the follow-up period in 2018, he or she was included in the study under our definition of a patient with new-onset arthritis.

2.4 Assessment of covariates

The covariates considered comprised sociodemographic characteristics, lifestyle behaviors, and current health conditions. Sociodemographic attributes encompassed age (in years), gender

(male/female), marital status, educational attainment (Elementary school and below/high school and college and higher), sleeping duration, and residential area (rural/urban). Lifestyle behaviors encompassed smoking habits (never smoking/former smoking and current smoking) and drinking status. Present health issues (yes/no) comprised hypertension and diabetes. Laboratory test results included triglycerides, glucose, and body mass index (BMI).

The diabetes diagnosis follows the criteria set forth by the American Diabetes Association in 2005 [14]. Diabetes was characterized by a fasting blood glucose level of ⩾126 mg/dl (7 mmol/L), and/or a random blood glucose level of ⩾200 mg/dl (11.1 mmol/L), and/or an HbA1c level of ⩾ 6.5%, and/or a self-reported confirmation in positive response to the question, "Have you ever been diagnosed with diabetes or hyperglycemia?". In addition, our classification of BMI follows the Chinese adult standards [15]. Underweight was characterized by a BMI below 18.5, normal weight ranged from 18.5 to 23.9, overweight was classified between 24 and 27.9, and obesity was indicated by a BMI of 28 or higher.

2.5 Statistical Analysis

The data was sourced from the CHARLS survey conducted between 2011 and 2018. Our investigation encompassed 4,418 participants. For continuous variables, the data were expressed as either the mean (standard deviation, SD) or median (interquartile range, IQR) and for categorical variables, as percentages. We employed a multivariable logistic regression model to scrutinize the relationship between the TyG index and new-onset arthritis. Stratified multivariate regression analysis was applied for subgroup evaluations. An interaction analysis was conducted to determine whether sociodemographic and health-related factors moderated the association of the TyG index with arthritis.

Moreover, the dose-response relationships between the TyG index and new-onset arthritis were visualized using restricted cubic splines with 4 knots at the 5th, 35th, 65th, and 95th percentiles. Finally, we used decision trees to identify high-risk populations for new-onset arthritis. We employed the Rpart program, integrated within the R environment, for the construction of decision trees. This facilitated the illustration of classification rules derived via recursive partitioning. In the process of tree development, we ensured equal misclassification costs for the different categories of the response variable[16].

Statistical analyses were conducted using R version 4.1, employing the 'ANOVA' function in the rms package for conducting restricted cubic spline (RCS) analysis, while decision tree models were generated using the 'Rpart' package. Statistical significance in the analysis was indicated by a two-tailed P-value below 0.05.

3. Results

3.1 Characteristics of the study participants according to the new-onset arthritis and TyG quartiles

Table 1 shows the attributes of participants involved in the study. Our finalized cohort comprised 4,418 participants, with 396 manifesting new-onset arthritis. In comparison to individuals devoid of arthritis, a greater proportion of women presented with arthritis, accompanied by lesser educational attainment, a reduced prevalence of hypertension, yet elevated levels of triglyceride and TyG. About the TyG quartiles, we found that elevated TyG levels significantly correlated with a

higher likelihood of female participants, increased prevalence of hypertension, diabetes, elevated glucose, TG, diminished HDL-C, augmented LDL-C, and enhanced CRP levels (See Table 2).

3.2 Relationship between TyG index and new-onset arthritis

Table 3 presents the results obtained from conducting multivariate regression analyses. The result shows that in comparison to the Q1 group, the incidence rate of arthritis surged by 40% in the last TyG index quartile group (Q4) within Model 2 (OR, 1.40; 95% CI, 1.03, 1.90) and by 39% in Model 3 (OR, 1.39; 95% CI, 1.01, 1.91). Figure 2 shows the findings from the Restricted Cubic Splines (RCS) assessment. It revealed that, within the context of the fully adjusted model, no dose-response relationship between the TyG index and arthritis was identified ($P_{overall}$=0.068, $P_{non\text{-}linear}$=0.203).

3.3 Stratified analysis

To assess the stability of the positive association between the TyG index and new-onset arthritis, participants were categorized into subgroups according to their socio-demographic variables and disease history, and the association was analyzed within each group (See Figure 3). Further interaction tests reveal that BMI may play a moderating role in the TyG index's association with new-onset arthritis (*P* for interaction =0.035). Individuals with a BMI ranging from 18.5 to 24 show an increased risk of developing arthritis associated with higher levels of the TyG index. Specifically, for individuals with a BMI ranging from 18.5 to 24, the TyG index for each unit increase is associated with a 64.0% (95% CI: 27.0%-112%) higher odds of arthritis, but there is no association for others.

3.4 Decision tree analysis

In Figure 4, the decision tree results for new-onset arthritis are illustrated. The root node of the model indicates that the incidence of arthritis is influenced by factors such as education level, TyG index, hypertension, and diabetes. From our analysis, the accuracy result of DT models is 80.42%, and two predominant high-risk subgroups for incident arthritis are identified. The first subgroup encompasses individuals who have not received a college education or higher, with a TyG index of 8.1 or greater, and without hypertension. Conversely, the second subgroup consists of those lacking a college or higher education, possessing a TyG index of 8.1 or above, but presenting with both hypertension and diabetes.

4. Discussion

The focus of cutting-edge research for chronic diseases may have shifted from improving treatment efficacy to primary prevention of the disease, as once formally diagnosed, it may be incurable for life. Therefore, from the perspective of preventing the occurrence of arthritis, utilizing population-based approaches to identify modifiable risk factors presents an 'opportunity window' for reducing the incidence and alleviating societal burden [17, 18]. Our longitudinal study found that an elevated TyG index increases the incidence of arthritis in individuals aged 45 and above in the overall population. Furthermore, the subgroup analysis provides additional supportive evidence for this conclusion, indicating that individuals within a normal BMI range are significantly affected by this association. Therefore, our findings not only expand the application scope of the TyG index but also provide strong evidence for its use as a detection method for arthritis prevention.

As a readily available and easily calculated metric, the TyG index has demonstrated its superiority over other indices such as HOMA-IR in assessing individual insulin resistance levels.

Irrespective of diabetic status or insulin treatment, the TyG index consistently exhibits robust sensitivity and specificity [19]. Currently, the application of this index has progressively expanded to encompass large-scale clinical investigations on T2DM, cardiovascular disease, pulmonary disorders, hepatic ailments, and various other medical conditions [20-23]. In the mechanism study of arthritis, researchers have discovered a strong correlation between IR and the occurrence of arthritis [6, 24]. For instance, there is a strong association between positive serum results for rheumatoid factor, anti-citrullinated protein antibodies, and IR in cohorts with early inflammatory polyarthritis [25]. In targeted therapy for RA, symptom improvement is associated with TC/HDL-C, and long-term follow-up results indicate a more substantial long-term improvement in IR [26]. Moreover, the shift in understanding the pathogenesis of OA from being solely attributed to joint wear to one caused by metabolic syndrome-induced systemic low-grade inflammation theory emphasizes the crucial role of IR in its early onset [27]. Further research has found that the TyG index can serve as a reliable screening method for IR in individuals diagnosed with immune-inflammatory conditions like RA and systemic lupus erythematosus [28]. It has been observed that RA patients demonstrate a higher incidence and severity of IR [29]. The TyG index evaluates IR by considering two closely associated risk factors: lipid metabolism and glucose metabolism. Impaired glucose metabolism is directly linked to the occurrence and progression of arthritis, making it a predictive risk factor not only for individuals with typical arthritic features but also for accelerated development [30]. Consequently, certain medications targeting arthritis, such as TNFα antagonists and interleukin-1β antagonists, exhibit potential effects on glucose metabolism by modulating glycolysis and oxidation[6]. These pharmacological interventions can alleviate abnormal osteoblast differentiation and joint cartilage damage [31, 32]. Furthermore, a compelling epidemiological association exists between dyslipidemia and arthritis. A clinical study revealed a positive correlation between the severity of knee osteoarthritis and elevated levels of triglycerides and total cholesterol [33], suggesting that higher levels of triglycerides and total cholesterol are associated with increased severity of knee osteoarthritis. Another retrospective analysis demonstrated significant disparities in lipid metabolism between newly diagnosed arthritis patients and the control group, with triglycerides exhibiting the most notable distinction by being 17% higher in those with arthritis compared to those without [33]. However, population-based studies have also indicated that isolated impaired glucose metabolism and specific lipid abnormalities appear unrelated to the occurrence of arthritis [34-36]. Hence, these simplistic associations fail to fully elucidate the findings presented in this study.

The biological mechanism of the correlation between the TyG index and arthritis risk remains elusive, it can be elucidated from an IR perspective and its associated mechanisms. Firstly, immune cell function relies on glucose metabolism, and insulin indirectly regulates immune responses systemically as well as in cartilage and synovial tissues through its hypoglycemic effects [37]. When IR develops, elevated blood glucose levels can lead to what is commonly referred to as "glucose toxicity. This condition induces cellular stress, promotes the production of advanced glycation end products, generates reactive oxygen species, and triggers the release of inflammatory cytokines [38]. In human chondrocytes, IR reduces autophagic flux while increasing Akt phosphorylation of autophagy inhibitory factor and synthesis of rpS6 regulatory protein, resulting in impaired chondrocyte autophagy that hampers removal of damaged intracellular metabolites [39]. In fibroblast-like synoviocytes (FLS), an experiment confirmed that insulin significantly inhibits matrix

metalloproteinase (MMP) 1, MMP13, ADAMTS4 in FLS from non-diabetic individuals exerting a protective anti-inflammatory effect [40]; however, IR significantly impairs this protective mechanism. In addition, the PI3K/mTOR/Akt/NF-κB signaling pathway is stimulated by increased insulin levels, leading to the generation of pro-inflammatory cytokines in FLS, while inhibiting cellular autophagy [41]. This suggests that high insulin can exacerbate inflammation in synovial cells either independently or through different signaling pathways. Abnormal glucose metabolism, a related issue derived from IR, is also associated with the early onset of arthritis. Disruption of the glycolysis and oxidative phosphorylation equilibrium occurs in the FLS of RA due to an elevation in glycolysis activity [42]. Experimental evidence suggests that invasive FLS exhibit significantly enhanced glucose metabolism, thereby contributing to joint damage in the arthritis model. Key enzymes involved in glycolysis, such as phosphoglycerate kinase, play important roles in promoting an inflammatory environment within joints and synovial hyperplasia during FLS invasion behavior [43-45]. Recent studies have shown a strong correlation between the synovial expression of IL-34 and factors such as rheumatoid factors, erythrocyte sedimentation rate, CRP, and the progression of arthritis on radiographic imaging [46]. Furthermore, IL-34 has been found to induce arthritis by promoting glycolysis expansion [47]. The augmented glycolysis induced by IL-34 results in the emergence of inducible nitric oxide synthase-positive macrophages, thereby enhancing fibroblast and Th1/Th17 cell polarization capabilities. This interplay between these two cell types amplifies inflammation and metabolic phenotypes, consequently accelerating arthritis progression through its impact on cellular energy metabolism, osteoclast formation, and neovascularization. Additionally, diabetic mice exhibited decreased expression of glucose transporter protein Glut1 along with reduced glycolytic rates. Moreover, specific induction of Glut4 deficiency further expedites cartilage loss in OA [48]. Collectively, these findings underscore the significance of improving glucose metabolism for delaying arthritis development.

Abnormal lipid metabolism is another important mechanism of IR, which initially results in excessive fat accumulation within the body. Adipose tissue, as the main source of inflammatory factors, chemokines, and metabolically active mediators, has a crucial impact on the development of arthritis [49]. Leptin and adiponectin, being important adipokines, not only regulate insulin sensitivity and lipid metabolism but also contribute to the occurrence of arthritis by modulating inflammatory and immune responses [50]. In the early stages of arthritis, when there is no apparent inflammation, individuals predisposed to arthritis experience a notable decrease in mitochondrial β-oxidation of long-chain fatty acids [51]. This suggests that changes in lipid metabolism occur before currently identified pathogenic mechanisms and may be the primary driving force behind arthritis. In the later stages of arthritis, the degree of inflammation in adipose tissue directly affects disease-related metabolic disturbances. Recent studies have shown a strong correlation between the presence of RA and the induction of an elevated inflammatory state in adipocytes, which is believed to be caused by the activation of intracellular kinases, as well as increased expression of IL1β and mTORC2. In experimental mice, observed changes include low levels of adiponectin and high levels of leptin, which are commonly associated with IR [52]. Research evidence suggests that lifestyles and dietary patterns associated with lipid metabolism significantly impact the occurrence and outcome of arthritis. Diet and exercise can ameliorate disease progression by mitigating inflammation response, and oxidative stress, and positively influencing gut microbiota [53]. We also observed a

pronounced influence of the TyG index on individuals with a normal BMI, aligning with existing research findings. One study demonstrated a stronger association between non-obese RA patients and systemic inflammation, disease activity, as well as IR development compared to obese patients [52]. Another study revealed that the RA group had an average HOMA-IR level 31% higher than the control group. Even more surprising, remarkably within a normal BMI range among RA patients, this average HOMA-IR level was discovered to be 61% higher compared to the reference range for healthy individuals [54]. This finding may be attributed to the susceptibility of individuals with a normal BMI to insulin resistance and dysregulation in glucose and lipid metabolism, underscoring the significance of maintaining optimal body weight.

4. Study strengths and limitations

Given the distinctive characteristics of CHARLS data, our study offers valuable supplementary insights from various perspectives [13]. Firstly, employing a prospective cohort study design strengthens the evidence and allows for partial causal inference regarding the TyG index as an independent risk factor in predicting incident arthritis. Secondly, our study data fills the gap in the population aged 45 and above, particularly representing Asian populations with Chinese individuals. The utilization of the TyG index as a predictor for new-onset arthritis risk presents evident advantages: it not only entails minimal screening costs but also enhances individual and physician motivation. Despite potential drawbacks such as overdiagnosis, early-stage disease modulation exhibits a favorable risk-benefit ratio considering the broad spectrum of disease risks among individuals with IR.

However, to facilitate the acceptance of meaningful supplements in future studies, it is imperative to provide a comprehensive elucidation of the limitations inherent in this investigation. Firstly, diet types and medications, such as statins and beta-blockers, significantly influence TG levels. Regrettably, due to insufficient data availability, we were unable to make targeted adjustments within our model. Similarly, an exhaustive analysis of specific subtypes of arthritis could not be conducted owing to the absence of clear classifications for arthritis diagnoses reported by patients themselves in CHARLS. Consequently, this study underscores the need for additional mechanistic research to complete the observed associations, providing a deeper understanding of the underlying processes. In addition, the participants selected for this study were relatively young, and compared to the general population aged 45 and above, they had a lower prevalence of diabetes or hyperlipidemia. Additionally, the outcomes of newly developed arthritis were determined based on self-reporting by individuals. This method may underestimate the actual data. These selection biases and information biases could potentially affect the representativeness of the study sample. Therefore, any findings from this study should be interpreted with caution.

5. Conclusions

The TyG index exhibits significant clinical value in predicting the risk of new-onset arthritis and serves as an independent risk factor that increases the likelihood of developing arthritis among individuals aged ⩾45 years in China's general population. This study underscores the significance of enhancing IR and addressing related metabolic abnormalities to prevent and delay the onset of arthritis, which may be critical for enhancing primary prevention efforts against this condition.

Triglyceride_glucose_index_in_the_prediction_of_new_ons...

ORIGINALITY REPORT

8%
SIMILARITY INDEX

PRIMARY SOURCES

1	www.frontiersin.org<br>Internet	57 words — 1%
2	www.ncbi.nlm.nih.gov<br>Internet	44 words — 1%
3	www.mdpi.com<br>Internet	35 words — 1%
4	assets.researchsquare.com<br>Internet	30 words — 1%
5	Chao Yang, Qinqin Meng, Huaiyu Wang, Yafeng Wang et al. "Anemia and Kidney Function Decline among the Middle-Aged and Elderly in China: A Population-Based National Longitudinal Study", BioMed Research International, 2020<br>Crossref	19 words — < 1%
6	Xin Gong, Yanyan Yu, Qing Tong, Ying Ren, Zhe Jin. "Effects of “Bu Shen Huo Xue Decoction” on the Endometrial Morphology and Expression of Leukaemia Inhibitory Factor in the Rat Uterus during the Oestrous Cycle", Evidence-Based Complementary and Alternative Medicine, 2013<br>Crossref	18 words — < 1%
7	mdpi-res.com<br>Internet	

18 words — < 1%

8	psj.gau.ac.ir<br>Internet	17 words — < 1%
9	Bokun Kim, Keisuke Taniguchi, Tomonori Isobe, Sechang Oh. "Triglyceride-glucose index is capable of identifying metabolically obese, normal-weight older individuals", Journal of Physiological Anthropology, 2024<br>Crossref	15 words — < 1%
10	atm.amegroups.com<br>Internet	15 words — < 1%
11	journals.plos.org<br>Internet	15 words — < 1%
12	www.dovepress.com<br>Internet	15 words — < 1%
13	www.science.gov<br>Internet	13 words — < 1%
14	link.springer.com<br>Internet	12 words — < 1%
15	journals.sagepub.com<br>Internet	10 words — < 1%
16	ecerm.org<br>Internet	9 words — < 1%
17	vdocuments.mx<br>Internet	9 words — < 1%
18	worldwidescience.org<br>Internet	

9 words — < 1%

19 Zayas, Janet P.. "Molecular Dissection of Impaired Infection Control in Diabetic Wound.", Rush University, 2020
ProQuest

6 words — < 1%

EXCLUDE QUOTES	OFF	EXCLUDE SOURCES	OFF
EXCLUDE BIBLIOGRAPHY	OFF	EXCLUDE MATCHES	OFF
